# Hierarchicality of Trade Flow Networks Reveals Complexity of Products

**DOI:** 10.1371/journal.pone.0098247

**Published:** 2014-06-06

**Authors:** Peiteng Shi, Jiang Zhang, Bo Yang, Jingfei Luo

**Affiliations:** 1 School of Systems Science, Beijing Normal University, Beijing, China; 2 Ministry of Commerce of the People's Republic of China, Beijing, China; National Scientific and Technical Research Council (CONICET)., Argentina

## Abstract

With globalization, countries are more connected than before by trading flows, which amounts to at least 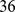 trillion dollars today. Interestingly, around 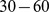 percents of exports consist of intermediate products in global. Therefore, the trade flow network of particular product with high added values can be regarded as value chains. The problem is weather we can discriminate between these products from their unique flow network structure? This paper applies the flow analysis method developed in ecology to 638 trading flow networks of different products. We claim that the allometric scaling exponent 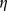 can be used to characterize the degree of hierarchicality of a flow network, i.e., whether the trading products flow on long hierarchical chains. Then, it is pointed out that the flow networks of products with higher added values and complexity like machinary, transport equipment etc. have larger exponents, meaning that their trade flow networks are more hierarchical. As a result, without the extra data like global input-output table, we can identify the product categories with higher complexity, and the relative importance of a country in the global value chain by the trading network solely.

## Introduction

As the process of globalization accelerates, countries in the world are more connected and collaborative unprecedentedly under the background of an integrated global markets of capital, labor force and products. Consequently, some cross-border production chains, which comprise several countries or regions, emerged inevitable as the result of international labor force division and collaboration in the global level [1]–[3]. However, due to the heterogeneities of products, the production networks are very inhomogeneous. Some products in the electronics and automotive industries, say PCs or automobiles, can be broken down into several independent components, and easily transported and assembled in different countries [1]. Therefore, a large fraction of imports for these products are not for final consumption but re-production with higher value-added and exports [1], [4], [5]. On the other hand, the networks for agriculture or raw material products may have much shorter production chains. Thereafter the major imports of these products are for final consumption.

Differentiating these products according to their production chains and level of added-values is of importance for countries' long term development strategy. Conventional method [6]–[8] tries to build the value flow networks among different products directly by incorporating the international input-output tables [9]–[11]. Although the whole picture of production networks can be captured in detail, obtaining the accurate raw data on the global level is not easy [8], [10]. On the other hand, the highly detailed international trade flow data for various products among countries are well documented for a long history [12], [13]. Particularly, all the bilateral trade flows are classified by different products according to the SITC (Standard International Trade Coding) or other equivalent coding methods. Therefore, a unique flow structure of one product category can be extracted from the international trade data.

World wide trade network as a specific instance of complex network has been studied for several years [14]–[16]. These early works always focus on country positions or node centralities on the network. Except network structure, recent works focus more on dynamics, weights and different trade networks by products. The longitudinal studies of trade networks reveal how the network structure such as the centrality of entire network changes along time to reveal the potential influences of globalization [17], [18]. Weights standing for trade flows between countries hide important information which cannot be uncovered by network structure solely [19], [20]. If the kinds of products exported by countries are considered, the problem of a country's industrial structure and export strategy can be studied by a country-product bipartite network model [21]–[23]. In this paper, however, we take the consideration of network structure, flows and different products in the same time. We construct weighted multi-networks of different products [24] from the trade flow data. For each product, there is a unique flow network which can be used to reflect the characteristic of the product. Therefore, we can discriminate products on their level of complexity and value-added by identifying their unique trade flow structures. It can work because trade networks contain the information of global production networks - almost all the cross-border product flows in the global value chain are recorded in the international trade data. This analysis is done in several levels of products classification because the trade flow datasets provide the hierarchical classification information.

Our methodology is to compare the allometric scaling exponents among the flow networks of different products [25], [26]. The allometric scaling pattern is found to be ubiquitous for trees spanned by binary networks [26], [27], like food webs [27], [28], trade webs [29] and biological networks [30]. Our previous work has incorporated the flow analysis methods developed in ecology to reveal the common nature of the flow networks in general [28], [31]. It is natural to extend this method to trade flows, in which, the allometric scaling exponent is given a new explanation, the degree of hierarchicality. It characterizes whether the product flows along a long hierarchical chain or not. We calculate the allometric exponent of each flow network in different product classifications, and find that the manufacture products with higher added values have larger exponents. Furthermore, most exponents are larger than one, meaning that the networks are hierarchical. While, the networks of the primary products with relative low added values have smaller exponents and the networks are flat. Hierarchicality always means inequality and monopoly. We further calculate the relative importance of each country in a product trading network, and compare the heterogeneities of country impact distribution for different products by GINI coefficient of country's impact. Finally, the dynamics of allometric scaling exponents along time is shown, and the globalization process can be read.

## Results

### Trade Flow Networks

We use two data sets to study and compare for eliminating the potential discrepancy from the data. The fist one is from Feenstra, et al's "World Trade Flows: 1962–2000" dataset based on the United Nations COMTRADE database (abbreviated by UN data set) [12](see Section 1 and Table S1 in File S1). This data set covers the bilateral trade flows of about 800 kinds of products according to the SITC 4 (Standard International Trade Classification system, Rev.4) classification standard from 1963 to 2000. And the results of 2000 year are mainly shown and discussed in the main text. Another data set (OECD data set) is the bilateral trade data in 2009 which was complied by the Organization of Economic Co-operation and Development (OECD) [13](see Table S2 and S3 in File S1). The OECD data set contains only the OECD member countries so that the total number of countries is smaller than the UN data set. However these countries dominate about 90% trade volume in the world. The products classification standard of the OECD data set is ISIC Rev.3 (International Standard Industrial Classification of All Economic Activities, Rev.3), which is slightly different from the SITC 4 classification. Please see detailed discussions of the data sets in File S1.

The SITC4 codes are hierarchical, meaning that the categories with longer codes are sub-categories of the ones with shorter codes if they share the same prefix. For example, the product category 7 in SITC4 stands for the category of machinery and transport equipment, so this is a very generalized classification. While, 71, 72 are two sub-categories of 7 representing the power machinery product and vehicle respectively.

### Allometric Scaling of Trade Networks

For each product trade network, we can define an exponent 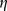 to characterize the hierarchicality of the flow network. At first, we need to calculate two vertex specific variables, namely, 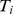 and 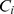.


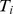
 called the trading volume of country 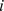, is defined as the maximum of 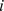
's total import or export. It reflects the capacity of trade flows through 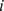. Next, 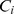 is the impact of 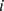 on the entire network. It is defined as the total changes of trading volume of other nodes on the network after the hypothetical delete of 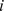. The concrete calculation of these two variables are referred to the method section.

**Figure 1:**
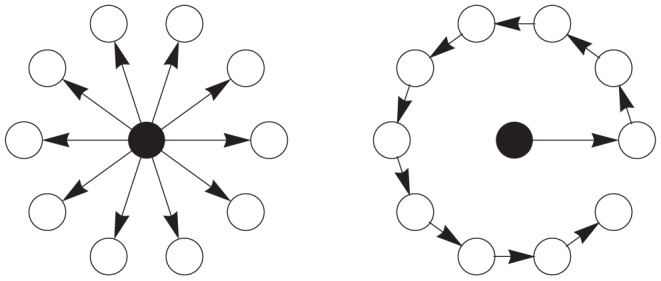
Two special spanning trees with minimum allometric exponent 1 (left, a star network) and maximum exponent 2 (right, a directed chain).

Usually, for various empirical trade networks, 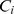 and 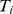 have a strong correlation which can be described by a power law, 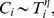
(1)where, 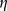 is the allometric scaling exponent. This equation is extended from the empirical allometries from river basin, vascular networks and food webs [26], [27]. The previous studies on spanning trees show that the exponent 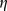 can be used to reflect the hierarchicality or flatness of a tree. For example, two extreme cases of spanning trees can be shown in Figure 1. The star network which has the smallest exponent 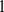 is the flattest tree, while the chain network which has the largest exponent 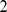 is the most hierarchical tree.

This calculation can be extended to general flow networks [28], [31], nevertheless the exponent is not bound in 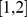. However, we can also define the exponent as the hiearchicality of a general flow network. Because it will contain long flow chains if its exponent is larger (see method section).

**Figure 2:**
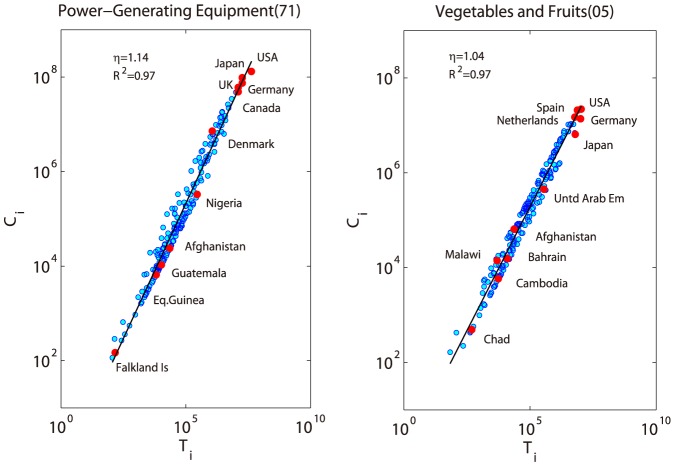
The allometric scaling law between 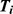 (in U.S. dollar) and 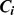 (in U.S. dollar) of two networks are shown. The left figure shows a super-linear scaling law (with exponent larger than 1) for power generating product, while the right one shows a sub-linear scaling law (with exponent smaller than 1) for fruit and vegetable.

It turns out that the allometric scaling pattern (Equation 1) is very general for all the studied trade networks in all classification levels but their exponents are not similar. Figure 2 shows the allometric scaling patterns of two products in two digits level: power-generating equipment (SITC4 code: 71) and vegetables & fruits (SITC4 code: 05).

In Figure 2, each data point stands for a country participating the international trade of this product. The pairs of 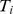 and 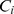 form a straight line on the log-log coordinate which means a power law relationship between the two variables exist (i.e., Equation 1). The exponents for these two products are distinct indicating that the power generating trade network is more hierarchical than the network of fruit and vegetable. In another word, the production for power generating machines is along a longer value-added chain than the fruit and vegetable.

**Figure 3:**
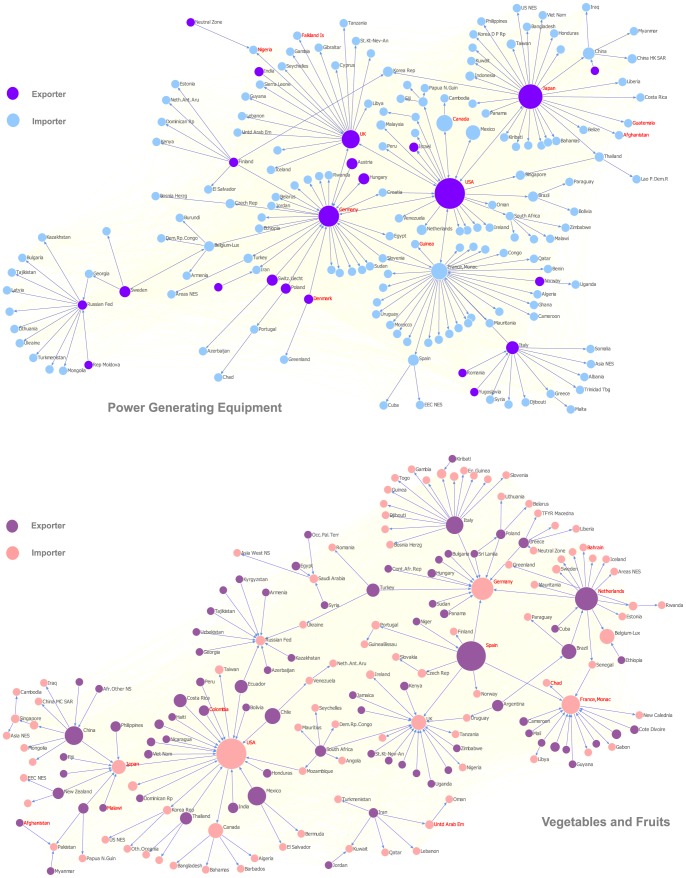
Visualization of trade flow network for power generating equipment (upper) and fruit and vegetable (lower). We use different colors to distinguish nodes as importer (import is larger than its export) and exporter (export is larger than import). The size of node denotes the total volume of trade. In these two networks, only the backbones are shown as the main parts and all other un-important links are hidden as backgrounds. The backbone extracting method is according to [35].

This point can be visualized by the network plots of these two products shown in Figure 3. Although only the backbone links are shown and other links are faded as backgrounds, it is clear that the upper network has many long chains which always root from some major exporters of power generating machine(e.g. U.S. and Japan). However, the lower network is more fragmental. Although several large countries (e.g. U.S.) still occupy a large fraction of fruit trade, most of them are importers. That implies the whole network is lack of center and more flat. Intuitively, that is the reason why the exponent of the first network is larger than the latter one.

### Exponents Comparison and Distributions

We further compare the exponents among different networks of products in the coarse classification level in a more systematic way. In Table 1 and 2, we list exponents for all 1-digit products in UN data set and OECD data set to compare.

**Table 1:** Exponents of 1-digit SITC4 categories in UN data set.

Code	Classification	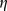	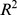	GINI
7	Machinery and transport equipment	1.136 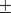 0.026	0.974	0.889
6	Manufactured goods classified chiefly by materials	1.120 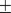 0.026	0.962	0.830
5	Chemicals and related products	1.117 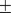 0.034	0.972	0.877
1	Beverages and tobacco	1.116 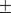 0.033	0.958	0.868
4	Animal and vegetable oils, fats and waxes	1.077 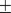 0.029	0.973	0.847
0	Food and live animals	1.043 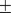 0.032	0.971	0.798
3	Mineral fuels, lubricants and related materials	1.042 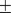 0.018	0.954	0.821
2	Crude materials, inedible, except fuels	1.001 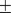 0.020	0.988	0.815
-	All Products	1.022 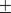 0.030	0.965	0.817

The categories of 8 (Miscellaneous) and 9(Not classified) are ignored in this table, The last row shows the allometry of all products as an integrated network.

**Table 2:** Exponents for different products in OECD data set.

Code	Classification	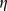	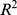	GINI
29	Machinery and equipment, nec	1.146 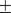 0.072	0.947	0.656
23T26	Chemicals and non-metallic mineral products	1.129 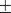 0.079	0.937	0.563
34T35	Transport equipment	1.124 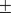 0.075	0.941	0.669
30T33	Electrical and optical equipment	1.112 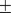 0.070	0.948	0.667
27T28	Basic metals and fabricated metal products	1.092 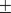 0.080	0.974	0.568
40T41	Electricity, gas and water supply	1.075 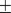 0.054	0.931	0.649
36T37	Manufacturing nec; recycling	1.074 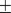 0.078	0.967	0.684
15T16	Food products, beverages and tobacco	1.073 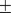 0.081	0.931	0.553
20T22	Wood, paper, paper products, printing and publishing	1.051 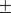 0.088	0.926	0.589
10T14	Mining and quarrying	1.019 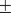 0.041	0.911	0.721
01T05	Agriculture, hunting, forestry and fishing	1.019 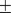 0.070	0.978	0.576
17T19	Textiles, textile products, leather and footwear	0.998 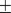 0.065	0.949	0.705
-	All industries	0.941 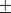 0.072	0.924	0.474

The products in different industries coded by ISIC Rev.3 coding system for industries is shown. Industries of financial intermediation, business services, wholesale and retail trade, transport and storage, post and telecommunication, hotels and restaurants, and construction are ignored because their trades do not stand for goods flows. The last row shows the allometry of all industries as an integrated network.

Both tables show large gaps of exponents for different products (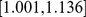 for UN-Comtrade data set and 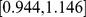 for OECD data set). Although some slight differences between SITC4 classification and ISIC Rev.3. classifications exist, the products of machinery, equipment, chemicals et al. are of higher exponents than the products of foods, mining and agriculture. This unique observation can be further confirmed and extended to finer classifications.

**Figure 4:**
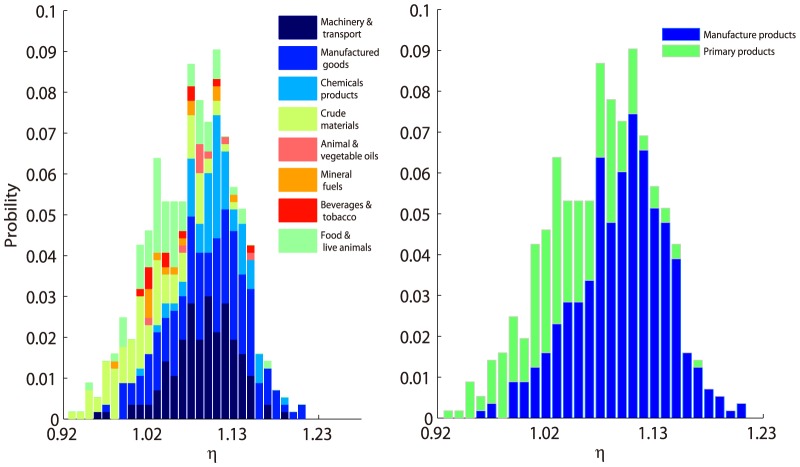
Exponents Distribution for All 4-digit SITC4 Product Categories. The stacked bar charts of different colors correspond to 1-digit SITC4 categories (left) and primary and manufacture classifications (right). For one specific 1-digit classification (say 0 for food and living animals), we can calculate the frequencies on each exponent intervals for all products with 0 prefix, then these frequencies as little bars are stacked on the tops of existing bars.

Figure 4 shows the exponents distribution of all products with 4-digits classification (the finest level in our dataset) in UN data set. The frequency curve has a bell-shape peaked at 1.09, which means most product networks are hierarchical. The stacked color bars show the distributions of all 1-digit classifications (Figure 4 left). Note that most blue bars locate in the right side of the bell-shaped curve, while, the green and yellow bars locate on the left side, indicating that the machinery and manufactured products have larger exponents than the food, beverage products. This phenomenon can be better illustrated by the right subplot of Figure 4, in which we simply classify the products as primary products (SITC4 codes prefix with 0,1,2,3,4) and manufacture products (SITC4 codes prefix with 5,6,7,8,9). The similar results can be derived for Leamer products classification standard (see SI section 3, Table S5 and Figure S2 in File S1).

### Allometric Exponent and Product Complexity

According to the observations, we know that the allometric exponents of trade flow network can reflect the basic properties of products. The manufacture products with higher added-value and complex production process always have larger exponents. Therefore, we conjecture that a positive correlation between the exponents and the nature of products (complexity or value added) may exist.

To test our hypothesis we do two correlation analysis on both data sets. For the UN data set, we correlate the exponents with PRODY, one of the measurements of product complexity. It is calculated as the average income level of the exporters (measured by the GDP percapita) of this product weighted by the comparative advantage of this product in different exportors [32]. It is calculated as: 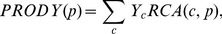
(2)where, 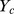 is the GDP per capita of country 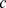, and 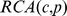 is the comparative advantage of country 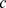 exporting 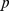. The summation is taken for all the countries exporting 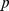. 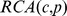 can be calculated as 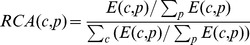, where 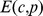 is the total export value of 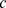 on 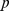. The numerator of the weight, 
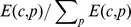
, is the value-share of the product 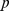 in the country 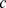
's overall export basket. The denominator of the weight, 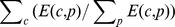, aggregates all the value-shares across all countries. Therefore, the weight measures the relative comparative of product 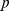 in country 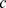. And 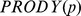 measures the average income level of 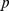. It is a proxy of the product's complexity.

**Figure 5:**
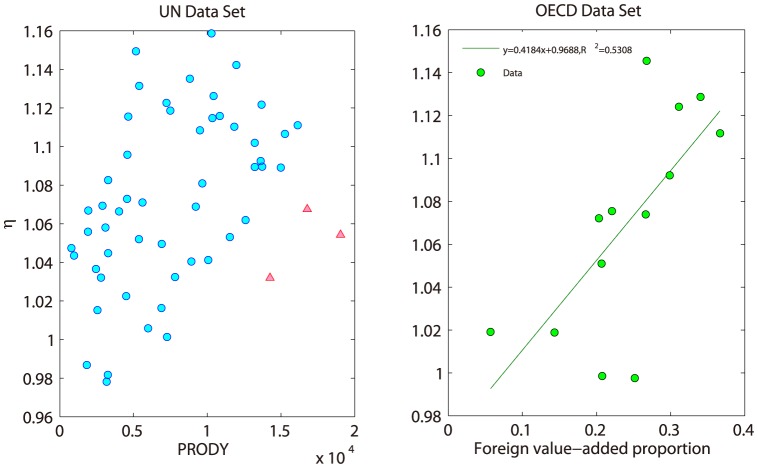
The relationship between 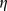 and PRODY of each 2-digit classification(left) in UN data set and 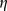 versus mean proportion of foreign value added for products in OECD data set.

Figure 5 shows the relationship between exponent 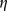 and PRODY of each product in 2-digits classification of UN data set. The correlation coefficient of these two variables is 0.37 and it can be improved to 0.44 if the three outliers (triangles) in Figure 5 are omitted.

For the OECD data set, the domestic and foreign value-added for each product-country combinations are available (see the discussion in Section 2 and Table S4 in File S1). This enables us to correlate exponents with average foreign value-added ratio of each product. Here, the proportion of foreign value-added is the ratio between the total value-added and gross export for all countries that exporting this product [1]. The relationship between 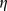 and foreign value-added proportion is shown in the right plot of Figure 5. There is a clear positive correlation between them, and the correlation coefficient is 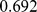.

Consequently, we conclude that the allometric exponent 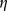 of each trade flow network can characterize the complexity and value-added proportion of given product. When a product needs more complex production processes, more countries must be involved to form a long value chain, so that more value is added on the product. All of these properties must be reflected in the flow structure of the product trade network. That is the reason why allometric exponent 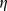 can be distinct for different products.

## Discussion

### Country Impacts

Besides the structural properties of the entire network, node positions in the global value chain are also of importance and interests. In our study, 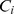, the total impact of country 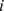 toward the entire network, can be viewed as a vertex centrality indicator because it measures the degree of the entire network is influenced if node 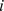 was removed. This understanding is in accordance with the standard HEM (Hypothetical Extraction Method) [33], [34] in input-output analysis once the trade flow networks are understood as an input-output matrix.

**Figure 6:**
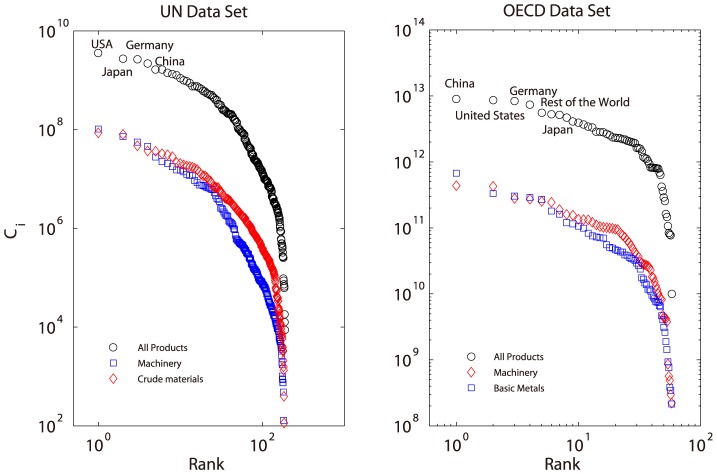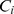 Distributions of Both Data Sets. The unit of 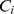 is U.S.dollar.

Figure 6 shows the distributions of 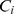 for trade networks of all products and several selected products both in UN and OECD data sets. Also, top 10 countries are listed in Table S6 and S7 in File S1.

### Centrality and Inequality

In our previous works of allometric scaling on ecological flow networks [31], the exponent 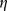 is explained as the degree of centrality, i.e., whether several big nodes dominate a disproportional impact on the entire network. This explanation can also be extent to this study. The networks with higher 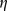
s are more centralized. So, a few large countries can impact the entire network, in which the impact's degrees 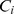 are disproportional to their direct trade flow 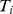.

For example, we have three flow networks with same 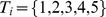 but different 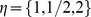. Then, their 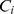 s are 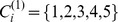 for 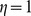, 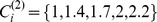 for 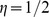 and 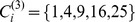 for 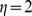 respectively. As a result, the largest country (the node with largest 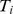) dominates 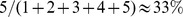, 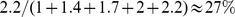 and 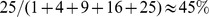 impacts of the entire networks respectively. Therefore, the third network is much more centralized than the second one.

However, the inequality of exporting products is mainly from the heterogeneity of the resource distribution but not the network effect which is characterized by 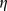. For example, petroleum export is heterogenous due to the unevenness of fossil fuel resource distribution geographically. Therefore, new indicator is needed.

We use the GINI coefficient of 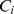 distribution to characterize the overall inequality of the flow network structure. 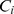 distribution can account for both inequality origins: natural resource distribution and network effect. First, it is obvious that the natural inequality of resource distribution can be reflected by 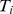 distribution. Suppose 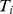 follows a Zipf law, 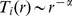, where, 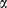 is the Zipf exponent, and 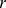 is the rank order of 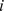. We know that there is a power law relationship between 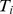 and 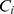 according to Equation 1. Thus, 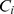 also follows the Zipf law: 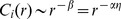, where 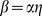 is its exponent. Therefore, the distribution of 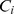 (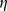) contains both information: natural heterogeneities (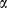) and network effect(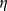).

Although 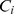 does not follow the Zipf distribution in our empirical data (shown in Figure 6), the previous conclusion that the distribution of 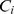 contains both information, is still correct. Usually, GINI coefficient (bounded by [0,1]) can be used to characterize the inequality of a variable no matter what kind of distribution it follows.

In the last column of Table 1, we show the GINI coefficients of all 1-digit product categories. Most products have similar rank order by GINI as the order by 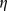. But the order of manufactured goods (Code 6) falls down from No. 2 (by 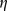) to No. 5 (by GINI coefficient), and the order of Food and live animals falls down from No.6 to the bottom. That indicates that these two kinds of products are not so unequal as predicted by the exponent 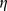 because the average trading volumes (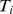) distribute evenly among countries although their trading networks are more centralized. In the last column of Table 2, the GINI coefficients of all industries of OECD data set are shown. There is a large deviation of the orders by 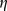 from the GINI coefficients. Some industries like mining and textiles have high ranks of GINI coefficients but low ranks of 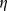. That means these industries are resource monopolized. While basic metals and chemicals have high ranks of 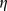 but low ranks of GINI coefficients which means the trade networks of these products are centralized.

Another interesting finding is the exponent of the integral trade network that consists of all trading products is 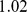 (It is 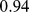 in OECD data set). This value is less than the mean exponent by averaging all individual products. It can be also observed for GINI coefficients. That implies international trade of all products in general becomes much more decentralized than each single product's trade. Therefore, trade on diverse kinds of products can make the world flatter. Though we still don't know in what degree this conclusion could be true. This will left for further investigation.

### Exponents in Different Years

**Figure 7:**
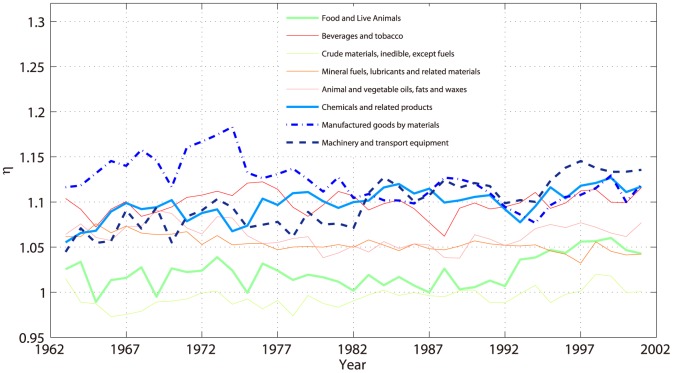
Allometric exponents 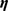 s of 1-digit classification products change with time.

The UN data set records the international trade data historically from year 1962 to 2000. This enables us to study the dynamics of exponents. In Figure 7, we show how these exponents change along time.

Most exponents are almost stable. However, machinery, transport equipment and manufactured goods by materials have big changes. The latter has very large exponents before 1982, but the former climbs to the top 1 after around 1982. Note that some cross-boarder companies emerged in around 1980s. Therefore, the product machinery and transport equipment which depends on vertical labor division but not material is of the largest exponent. While, the manufactured goods which is more independent on global cooperation change in an opposite direction. Hence, the dynamics of the exponents may reflect the globalization process.

## Methods

### Flow Network Model

A flow network model can be built for each product category. Nodes on the network are countries, directed edges are trading relationships between countries and weights on edges are trading flows measured by the unified money units (It is U.S. dollar in our data sets).

If there are totally 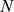 countries participating trade of the focus product 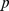, then a flow network can be represented by an 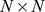 flux matrix 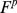, in which the element 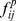 stands for the trade flow of 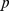 from 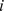 to 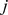. The superscript 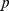 will be omitted to facilitate our expression. And all the variables as well as the trade networks in the following sections are defined for one specific product.

### From Trees to General Flow Networks

**Figure 8:**
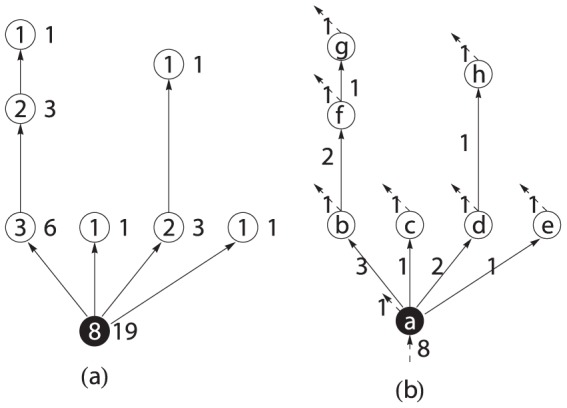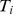 and 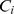 for trees. In (a), the numbers inside the nodes are 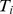
s and the numbers beside nodes are 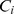
s. (b) is a flow network constructed according to (a), in which numbers represent flows. And dotted lines stand for dissipations.

Previous studies on network allometry can only be applied to directed trees. In which 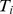 is the total number of nodes in the sub-tree rooted from 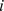 and 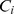 is the summation of all 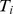
s in the sub-tree rooted from 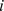 [26], [27] as shown in Figure 8 (a).

It is very difficult to generalize this definition for flow networks because the concept of sub-tree is vague due to the existence of loops. However, we can understand the directed tree as a flow network as shown in Figure 8(b) by assuming each node has one unit dissipation out of the network. Therefore, 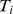 is just the flux through node 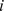. And 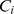 is the total flows reduced by the hypothetic removal of node 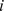. For example, if we remove node b in Figure 8(b), then all the flows in the sub-tree rooted from b disappear. The total amounts of these flows are 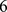. Therefore 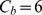. In this way, we can extend the definitions of 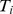 and 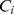 for general flow networks although the calculation of 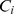 is not easy. The detailed discussion of this method can be referred to [28], [31].

### Trading Volume and Impact

In this subsection, we will show the method on computing 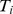 and 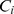 in detail. Firstly, 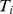 defined as the trading volume of country 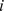, is the maximum value of either import or export, 
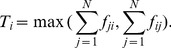
(3)


It measures the amount of product 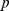 flows through country 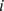. 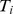 reflects the flow capacity that country 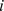 can import or export 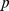.


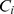
 is defined as the total reduction of trade volume of all countries if 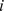 is deleted in the network. Although its definition is clear, the calculation is difficult. We will adopt the method of HEM (hyperthermic extraction method) [33], [34] in input-output theory to compute.

Before 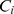 is defined, we should introduce another important matrix 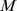 in advance. It is the analogy of technical coefficient matrix in input-output theory, 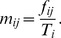
(4)


So, 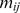 measures the ratio of the export from 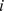 to 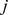 to the total trade volume of 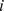. Then, the following identity can be derived 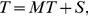
(5)where, 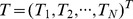, 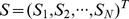. And 
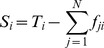
(6)can be viewed as the total domestic value-added from 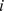 (see the discussion in SI section 2, Figure S1, S3 in File S1). Then, we can obtain an important identity from Equation 5: 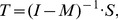
(7)where, 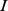 is the identity matrix. Now, suppose node 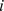 is deleted in the network, then the ith column in 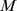, and also 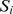 will be set to 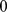 according to the HEM method [33], [34]. Suppose 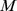 turns into 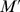 and 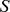 turns into 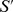. Then, the new total trade volume vector can be computed if we believe the identity Equation 7 is also hold for 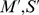 and 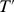: 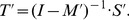
(8)


Then the total amount of trade volume reduction in the entire network is defined as 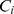, 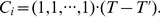
(9)


To ease our calculation, we always use the following equation 
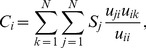
(10)where, 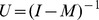. It can be proved that Equation 10 equals Equation 9 (see section 6 in File S1).

### Network Allometry

Allometric scaling is a universal pattern of transportation networks including rivers, vascular networks, etc. The allometric exponents for trees are bounded in between 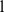 and 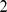. The minimum exponent can be obtained by a star-liked network, in which all links are from the root to other nodes, while, the maximum exponent is gotten by a chain as shown in Figure 1. These two special trees stand for two extremes for all directed trees. The star-liked tree is flat because every node except the root is equivalent. However, the chain-liked tree is hierarchical because the nodes in the upper level dominate the other nodes in the lower level.

According to the discussion in the previous sections and our previous works [28], [31], the network allometry is extended for general flow networks. Although the range of 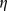 is not bounded to 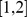, 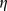 can be still a good indicator for the level of hierarchicality of the flow structure because the relative speed of 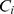 can increase faster than 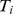 in a network with larger exponent. The network is more like a chain if its exponent is large. Therefore, some long flow chains can be revealed in these networks.

We distinguish networks as hierarchical (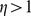), neutral (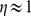) and flat (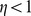) by the exponent.

## Conclusions

The most interesting finding of this paper is that the properties of a trade product can be reflected by the distinct flow structure of its trading network. Especially, the complexity or the level of value-added of a product can be characterized by the hiearchicality of the flow network which is measured by the allometric exponent. This conclusion is hold for different datasets in different coarse-grained levels of product classifications. Therefore, the information of production chain for different products and the relative positions of countries in the chain can be read from the international trade network.

## Supporting Information

File S1This file includes Table S1-Table S7 and Figure S1-Figure S3. Table S1, The dataset form in UN dataset. Table S2, The trade data in OECD dataset. Table S3, The value added data in OECD dataset. Table S4, The result of 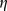 computed according to (4) and (5). Table S5, Exponents of Leamer Classification Standard. Table S6, The top ten 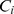 of different products in UN dataset. Table S7, Top ten countries of different industries in the OECD Dataset. Figure S1, Balanced value flow of one country. Figure S2, Exponents Distribution for All 4-digit Leamer Classification Standard. Figure S3, The relationship between 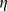 and the mean proportion of foreign value added.(DOC)
