# Supplementary material for: Hierarchicality of Trade Flow Networks Reveals Complexity of Products
Source: PLoS One. 2014 Jun 6;9(6):e98247. doi: 10.1371/journal.pone.0098247 (PMC4048167; doi:10.1371/journal.pone.0098247)
Supplement: File S1 — This file includes Table S1-Table S7 and Figure S1-Figure S3. Table S1, The dataset form in UN dataset. Table S2, The trade data in OECD dataset. Table S3, The value added data in OECD dataset. Table S4, The result of computed according to (4) and (5). Table S5, Exponents of Leamer Classification Standard. Table S6, The top ten of different products in UN dataset. Table S7, Top ten countries of different industries in the OECD Dataset. Figure S1, Balanced value flow of one country. Figure S2, Exponents Distribution for All 4-digit Leamer Classification Standard. Figure S3, The relationship between and the mean proportion of foreign value added. (DOC) [file pone.0098247.s001.doc]

### Supplementary Materials of “Hierarchicality of Trade Flow Networks Reveals Complexity of Products”

Peiteng Shi, Jiang Zhang, Bo Yang, Jingfei Luo

1. **Detailed information of Data Sources**
   1. **UN dataset**

The UN dataset records the highly detailed worldwide bilateral trade flows from 1962-2000. The dataset is available through the NBER-UN world trade database ([www.nber.org/data](http://www.nber.org/data)). SITC4 classifies all products in the dataset into approximately 800 different products. Table S1 shows a sample of the detailed data in the UN dataset.

In this table, importer and exporter stand for the countries of a transaction, icode, and ecode are their codes, respectively. Column SITC4 identifies the type of the product traded. Unit is the commodity unit, W is the weight (kilograms) and indicates the goods are measured by weight. There are also some commodities measured with V(volume), N(number of items) and so on. Dot indicates the source of data. A value of 1 stands for the data is from importer, whereas a 2 stands for data from an exporter. Value is the trade value measured by US dollars, whereas quantity is the amount of goods exported in different units. In this paper, we use the trade value to express the trade volume.

**Table S1. The dataset form in UN dataset**

| **year** | **Icode** | **importer** | **ecode** | **exporter** | **sitc4** | **unit** | **dot** | **value** | **quantity** |
| --- | --- | --- | --- | --- | --- | --- | --- | --- | --- |
| 2,000 | 218400 | USA | 537240 | Spain | 6577 | W | 1 | 257 | 28 |
| 2,000 | 218400 | USA | 537240 | Spain | 6579 | W | 1 | 2,998 | 406 |
| 2,000 | 218400 | USA | 537240 | Spain | 6581 | W | 1 | 445 | 125 |
| 2,000 | 218400 | USA | 537240 | Spain | 6582 | W | 1 | 132 | 10 |
| 2,000 | 218400 | USA | 537240 | Spain | 6583 |  | 1 | 3,532 |  |
| 2,000 | 218400 | USA | 537240 | Spain | 6584 | W | 1 | 18,297 | 1,986 |
| 2,000 | 218400 | USA | 537240 | Spain | 6589 |  | 1 | 4,940 |  |
| … | … | … | … | … | … | … | … | … | … |
| … | … | … | … | … | … | … | … | … | … |
| 2,000 | 331520 | Chile | 330760 | Brazil | 0483 | W | 1 | 281 | 159 |
| 2,000 | 331520 | Chile | 330760 | Brazil | 0484 | W | 1 | 698 | 349 |
| 2,000 | 331520 | Chile | 330760 | Brazil | 0577 | W | 1 | 178 | 27 |
| 2,000 | 331520 | Chile | 330760 | Brazil | 0579 | W | 1 | 391 | 293 |
| … | … | … | … | … | … | … | … | … | … |
| … | … | … | … | … | … | … | … | … | … |

**1.2 OECD dataset**

The OECD dataset is from the Organization for Economic Co-Operation and Development and can be found at ([http://stats.oecd.org](http://stats.oecd.org/)). The products classification is based on ISIC3 (International Standard Industrial Classification Rev. 3). ISIC3 classifies all products into 18 categories. This dataset only provides the detailed trade information of 57 OECD countries and regards other countries as the ‘Rest of the World’. In addition to the trade value of countries, it also includes the foreign value added content of gross exports and domestic value added embodied in gross exports.

Table S2 shows the structure of this dataset. The first column industry records the ISIC3 code of the trade. The dataset lists the partners and gross export (million dollars) for each country considered. The value added information of each country and industry is also provided in a separate table for this dataset. Table S3 shows the sample records of value added table. The domestic value added, foreign value added, and the gross export measured by million dollars are recorded in 18 industries and 57 countries. From the table, we can see the following:

*Gross Export = Foreign value added content in gross export + Domestic value added embodied in gross export.*

**Table S2. The trade data in OECD dataset**

| **Industry** | **Country** | **Partner** | **Gross Export** |  |
| --- | --- | --- | --- | --- |
| 10T14: Mining and quarrying | Canada | India | 109 | |
| 10T14: Mining and quarrying | Canada | Indonesia | 156 | |
| 10T14: Mining and quarrying | Canada | Latvia | 0.2 | |
| 10T14: Mining and quarrying | Canada | Lithuania | 0.1 | |
| 10T14: Mining and quarrying | Canada | Malaysia | 22.7 | |
| 10T14: Mining and quarrying | Canada | Malta | 0 | |
| … | … | … | … | |
| … | … | … | … | |
| 29: Machinery and equipment, nec | Chile | Malaysia | 0.5 | |
| 29: Machinery and equipment, nec | Chile | Malta | 0 | |
| 29: Machinery and equipment, nec | Chile | Philippines | 1.2 | |
| 29: Machinery and equipment, nec | Chile | China | 4.2 | |
| 29: Machinery and equipment, nec | Chile | Romania | 0 | |
| … | … | … | … | |
| … | … | … | … | |

**Table S3. The value added data in OECD dataset**

| **Industry** | **Country** | **Gross Export** | **Foreign value**  **added** | **Domestic value added** | |
| --- | --- | --- | --- | --- | --- |
| 10T14: Mining and quarrying | Belgium | 606.5 | 171 | 435.4 |  |
| 10T14: Mining and quarrying | Canada | 84512.6 | 5527.9 | 78984.7 |  |
| 10T14: Mining and quarrying | Chile | 26778.3 | 1427.4 | 25350.9 |  |
| 10T14: Mining and quarrying | Denmark | 3969.3 | 455.3 | 3514 |  |
| 10T14: Mining and quarrying | Estonia | 60.8 | 13.3 | 47.4 |  |
| … | … | … | … | … |  |
| … | … | … | … | … |  |
| 34T35: Transport equipment | Malaysia | 1506 | 713.3 | 792.8 |  |
| 34T35: Transport equipment | Malta | 125.8 | 41.4 | 84.4 |  |
| 34T35: Transport equipment | Philippines | 1261 | 434.3 | 826.7 |  |
| 34T35: Transport equipment | Romania | 5320.1 | 1397.2 | 3922.9 |  |
| … | … | … | … | … |  |
| … | … | … | … | … |  |

- 1. **The comparison of the two classification datasets**

The SITC4 and ISIC3 classification systems are connected to each other. They both obey the HS rule (Harmonized Commodity Description and Coding System). A detailed mapping between the SITC4 classification and ISIC3 classification is available from (http://mpra.ub.uni-muenchen.de/[27626/](http://mpra.ub.uni-muenchen.de/27626/) ).

The SITC4 and ISIC3 classification standards are designed for different purposes. SITC4 is the aggregated classification of transportable goods for international trade statistics, and it divides the goods into 10 main categories according to the types of goods. SITC4 does not include services.

ISIC3 mainly reflects the economic activities of a country, and it divides the products according to which activity the goods belong to: the first industry, second industry and third industry. Compared with the SITC4 classification, ISIC3 is not hierarchical.

Although several drawbacks exist with the OECD data, the data provide concrete information of added value, including foreign value added and domestic added in gross export for one specific industry. It is very important for our flow network balance analysis.

1. **Flow Network Analysis**
   1. **Construction of the product specific flow networks**

In this paper, we construct the flow network in this following manner. First, we select one type of product to study. Suppose this product’s code is XXX, then we select all of the records with code XXX as a subset of the records. Then, we construct a flow network with this subset of records. If the focus product is a category with code X, then we select all of the items with prefix X as the subset.

Once the subset of records is selected, the flow network can be built directly. Suppose there are *N* countries joining the trade of product *k* in the world, then we can use a flux matrix *F^k^* to describe the flow network as follows:

$F_{N\times N}^{k}=\{f_{ij}^{k}\}_{N\times N}$ (1)

where $f_{ij}^{k}$is the goods flow that country $i$exports to country$j$, measured by the total amount values of products.

- 1. **Value conservation and flow balance**

All of the analyses in this paper are based on the global value flow embodied by products in international trade. We assert that the value flows behind the flow network are conservative in general, which means values cannot be created or destroyed during the flow process embodied by trade. This proposition must be true because the mass is conservative.

However, we know that the trade flows are not balanced for most countries, i.e., their total import does not balance with the total export. If we consider the domestic production and consumption, the paradox can be avoided. The whole picture of one country’s trade flows should be depicted in the following ,ammer:

**Import**

**Production**

**Export**

**Consumption**

**Figure S1. Balanced value flow of one country**

In Figure S1, we know that the total input values must balance the total output values if the domestic production and consumption are considered, although they are not recorded in the international trade data.

Therefore, we can write down a flow balance equation:

$S_{i}+\sum_{j=1}^{N} f_{ji}=D_{i}+\sum_{j=1}^{N} f_{ij}$ (2)

where *S_i_*, *D_i_* are the domestic production and consumption of country *I,* respectively. $f_{ij}$ is the trade flow from *i* to *j*.

However, because the information on *S_i_* and *D_i_* is unavailable for most countries, we must manually estimate them.

The simplest method to estimate *S_i_* and *D_i_* is using the net export value. If the net export value of a country is larger than 0, then *D_i_=0, S_i_=*net export value, whereas if the net export value of a country is less than 0, then *S_i_=0, D_i_=*net export value. That is

$\left\{\begin{matrix} S_{i}=\max(0,\sum_{j} f_{ij}-\sum_{j} f_{ji}) \\ D_{i}=\max(0,\sum_{j} f_{ji}-\sum_{j} f_{ij}) \end{matrix}\right.$ (3)

Most results of main text are based on this naïve balance method and according to this, *T_i_* is just the total inflow or total out flow because of the following:

$T_{i}=\max(\sum_{j} f_{ij},\sum_{j} f_{ji})=S_{i}+\sum_{j} f_{ji}=D_{i}+\sum_{j} f_{ij}$ (4)

That is the reason why we call *T_i_* as the total trade volume of country$i$.

One may think this naïve method to estimate *S_i_* and *D_i_* is too simple to be correct. However, we will note that this method can lead to similar results toward the characterization of network hierarchicality ($\eta$) as the case when the empirical information of *S_i_* and *D_i_* is considered.

- 1. **Balancing the OECD trade network**

Fortunately, the information on every country’s domestic value added (*S_i_*) embodied in gross export is available for the OECD trade network. Therefore, *D_i_* can be inferred based on Equation (2):

$D_{i}=S_{i}+\sum_{j=1}^{N} f_{ji}-\sum_{j=1}^{N} f_{ij}$ (5)

Thus, a complete balanced value flow network can be obtained from the OECD dataset. Then *T_i_*, the total through flow should be of new form

$T_{i}=S_{i}+\sum_{j=1}^{N} f_{ji}=D_{i}+\sum_{j=1}^{N} f_{ij}\neq\max(\sum_{j=1}^{N} f_{ji},\sum_{j=1}^{N} f_{ij})$ (6)

The method for computing *C_i_* and $\eta$ is not changed. However, when we compare the ranking orders of all industries based on the exponent computed by this method and the naïve method, we obtain similar results (compare Table S4 and Table 2 in the main text).

**Table S4. The result of $\eta$ computed according to (4) and (5)**

| Code | Name | $\eta$ | $R^{2}$ |
| --- | --- | --- | --- |
| 29 | Machinery and equipment, nec | 1.097$\pm$0.052 | 0.979 |
| 34T35 | Transport equipment | 1.091$\pm$0.038 | 0.977 |
| 30T33 | Electrical and optical equipment | 1.085$\pm$0.037 | 0.971 |
| 23T26 | Chemicals and non-metallic mineral products | 1.064$\pm$0.043 | 0.969 |
| 27T28 | Basic metals and fabricated metal products | 1.062$\pm$0.048 | 0.978 |
| 40T41 | Electricity, gas and water supply | 1.055$\pm$0.049 | 0.977 |
| 36T37 | Manufacturing nec; recycling | 1.051$\pm$0.052 | 0.972 |
| 15T16 | Food products, beverages and tobacco | 1.047$\pm$0.050 | 0.966 |
| 10T14 | Mining and quarrying | 1.028$\pm$0.045 | 0.982 |
| 20T22 | Wood, paper, paper products, printing and publishing | 1.026$\pm$0.043 | 0.965 |
| 01T05 | Agriculture, hunting, forestry and fishing | 1.013$\pm$0.043 | 0.962 |
| 17T19 | Textiles, textile products, leather and footwear | 1.009$\pm$0.051 | 0.965 |
| - | All products | 0.999$\pm$0.102 | 0.990 |

From Table S4 and Table 2 in the main text, we know the ranking orders are all same except for the chemicals, mining and quarrying industries. However, the discrepancies among the exponents in Table S4 are much smaller than the ones in Table 2 of the main text. Therefore, we conclude that the naïve method of balancing a network can lead to better results in regards to distinguishing various industries.

1. **Exponent Distribution of Products Classified by Leamer Classification Standard**

**Table S5. Exponents of Leamer Classification Standard**

| **Code** | **Name** | $\eta$ | $R^{2}$ |
| --- | --- | --- | --- |
| 8 | Capital intensive | 1.129$\pm$0.029 | 0.968 |
| 10 | Chemicals | 1.117$\pm$0.027 | 0.972 |
| 9 | Machinery | 1.093$\pm$0.021 | 0.982 |
| 3 | Forest products | 1.083$\pm$0.025 | 0.975 |
| 7 | Labor intensive | 1.080$\pm$0.025 | 0.974 |
| 4 | Tropical agriculture | 1.064$\pm$0.030 | 0.963 |
| 6 | Cereals | 1.061$\pm$0.031 | 0.959 |
| 1 | Petroleum | 1.049$\pm$0.016 | 0.988 |
| 5 | Animal products | 1.031$\pm$0.027 | 0.966 |
| 2 | Raw materials | 1.014$\pm$0.019 | 0.983 |


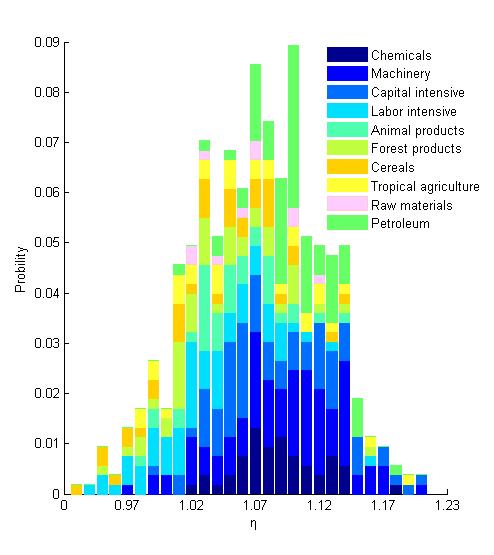


**Figure S2. Exponents Distribution for All 4-digit Leamer Classification Standard**

Leamer classification further divides the products coded by SITC4 into 10 categories according to the correlation of the products ---Petroleum, Raw materials, Tropical agriculture, Cereals, Forest products, Animal products, Labor intensive, Capital intensive, Machinery and Chemicals^[3]^. The Leamer classification system is considered more reasonable than SITC4 ^[2]^ because SITC4 is mainly for international trade and not according to the product’s own properties. Here, we demonstrated that the distribution of $\eta$ with the Leamer classification (Table S5 and Figure S2). Figure S2 shows the $\eta$ distribution of every product if they are corresponding to their subdirectories’ products which are coded in 4-digit codes.

From Table S5, the capital intensive products have the largest $\eta$, whereas the labor intensive products’ $\eta$ is lower. The Machinery and Chemicals are still on the top of the sorted$\eta$list.

1. **The relationship between $\eta$ and average value added if domestic production and consumption are considered**

**Figure S3. The relationship between $\eta$ and the mean proportion of foreign value added**

***(****The slope of the regressing line is 0.258;, the intercept is 0.984; the P value= 0.035;*$R^{2}$*=0.345 and the confidence interval is [0.022, 0.494])*

Figure S3 shows the relationship between $\eta$ and mean proportion of foreign value added in the OECD dataset if the domestic value-added information is considered to balance the network. The Pearson Correlation is 0.587. The *P* value is 0.035, and the correlation is significant at the 0.05 level (2-tailed). The Spearman Correlation is 0.731 and is significant at the 0.01 level (2-tailed).

Thus, this correlation can prove that the higher the$\eta$ is, the longer the product’s value-added chain is in another way. When comparing with $\eta$ without considering the value-added data, the correlation with the mean proportion of foreign value added is 0.629.

1. **List of Top Ten Countries sorted by** **C_i_ for different products**

*C_i_* is defined as the impact of country *i*. Therefore, the *C_i_* value can be understood as a type of vertex centrality. Here, we list the top ten countries of *C_i_* for different products.

**Table S6. The top ten C_i_ of different products in UN dataset**

|  | **Top1** | **Top2** | **Top3** | **Top4** | **Top5** | **Top6** | **Top7** | **Top8** | **Top9** | **Top10** |
| --- | --- | --- | --- | --- | --- | --- | --- | --- | --- | --- |
| Food & live animals | USA | Netherlands | France | Germany | Canada | Spain | UK | Japan | Brazil | Italy |
| Beverages & tobacco | France | USA | UK | Netherlands | Germany | Italy | Spain | Belgium | Mexico | Japan |
| Crude materials | USA | Canada | Australia | Germany | Brazil | Japan | China | Netherlands | Russian Fed | France |
| Mineral fuels  & lubricant | Saudi Arabia | USA | Russian Fed | Norway | Iran | Areas NES | Japan | Canada | Venezuela | UK |
| Animal & vegetable oils | Malaysia | Indonesia | Netherlands | Spain | USA | Italy | Germany | Argentina | Belgium | Canada |
| Chemicals | USA | Germany | France | UK | Ireland | Netherlands | Japan | Belgium | Italy | Switzerland |
| Manufactured goods | Germany | USA | Italy | Japan | China | France | UK | Belgium | Russian Fed | Canada |
| Machinery & transport | Japan | USA | Germany | China | UK | France | Korea Rep | Taiwan | Canada | Mexico |
| All Products | USA | Japan | Germany | China | France | UK | Canada | Italy | Netherlands | Korea |

Because different countries have comparative advantages in different products, the top 10 countries vary depending on product type.

We all know that Saudi Arabia is quite rich in fuel resources, such as petroleum. It is the largest exporter of petroleum. Thus it also has a large impact on the products related to oil. We can see that Saudi Arabia takes the first place in Mineral fuels & lubricant products, whereas Japan and America are quite famous for their cars, mechanical and electronic equipment. They have advanced technology and rich capital. Japan and the USA are the hubs of the Machinery & transport equipment trade,

Malaysia has a tropical rain forest climate, which is quite suitable for growing of plants and animals. Oil palm is a type of plant and has the most abundant oil of all types of oil crops. Malaysia is rich in the oil palm plantations and has a large impact on the oil palm trade of other countries. We can see from Table S6 that Malaysia is the most important country in the Animal & vegetable oil trades.

**Table S7. Top ten countries of different industries in the OECD Dataset**

|  | **Top1** | **Top2** | **Top3** | **Top4** | **Top5** | **Top6** | **Top7** | **Top8** | **Top9** | **Top10** |
| --- | --- | --- | --- | --- | --- | --- | --- | --- | --- | --- |
| Agriculture, hunting | USA | Rest | France | Brazil | Canada | Netherlands | China | Malaysia | Viet Nam | Spain |
| Mining & quarrying | Rest | Saudi Arabia | Russian Fed | USA | Norway | Canada | China | Australia | Japan | Germany |
| Food products | Netherlands | France | USA | Rest | Germany | Brazil | UK | Italy | China | Ireland |
| Textiles & textile products | China | Rest | USA | Italy | France | Turkey | India | Germany | UK | Thailand |
| Wood, paper, paper products | USA | Rest | Germany | China | Canada | France | Sweden | UK | Italy | Austria |
| Chemicals | Germany | USA | China | Netherlands | France | Rest | UK | Japan | Italy | Belgium |
| Basic metals  products | Germany | China | Japan | USA | Italy | Russian Fed | France | Rest | Korea  Rep | UK |
| Machinery & equipment | Germany | China | USA | Italy | Japan | France | Rest | Switzerland | UK | Korea |
| Electrical & optical equipment | China | USA | Japan | Korea Rep | Chinese Taipei | Germany | Thailand | Rest | Mexico | Singapore |
| Transport equipment | Germany | Japan | USA | France | Korea | UK | China | Mexico | Canada | Italy |
| Manufacturing | China | USA | India | Italy | Japan | Germany | Rest | France | UK | Poland |
| Electricity, gas & water supply | Switzerland | Germany | Russian Fed | Belgium | Netherlands | France | Canada | Italy | Austria | Czech Rep |
| Construction | Poland | China | Turkey | Belgium | UK | Russian Fed | Netherlands | Austria | Malaysia | Italy |
| All Products | China | USA | Germany | Rest | Japan | France | UK | Italy | Korea Rep | Netherlands |

*Rest in Table2 stands for the Rest of the World*

Table S7 lists the top ten *Ci* of different industries in ISIC3. We can find similar results in Table S6. Netherlands is the hub in the trade of Food products, whereas Germany, the USA and Japan control the trade of Machinery & equipment and Transport equipment.

*C_i_* can reflect the impact of one country to its direct and indirect partners and can be regarded as the importance of one country in international trade. Countries may have different impacts different trade markets.

1. **Computation of C_i_**

We can use two methods to compute *C_i_*. The first method is based on flowing particles.

We suppose that there are many particles flowing in the network, and those passing vertex $i$will be colored red. *C_i_* would then be the total number of red particles flowing in the network. The detailed explanation can be referred to this paper [1]. *C_i_* can be calculated as follows:

(1)

This method has been previously used by scholars^[1]^. However, it is hard to generalize these flowing particles picture to international trade flows because we know that flowing goods are not random particles.

Therefore, in this paper, we give a totally different method to define *C_i_*. It is defined as the total reduction of trade flows in the network if the concerned country *i* is deleted, i.e., the impact on the entire network of *i* (see the methods section in the main text). The interesting finding is these two methods can be proved to be equivalent.

This equivalence can be stated by a theorem (Theorem 1). Before the theorem is stated, we need to define a set of notations.

$S=(S_{1},S_{2},\cdots,S_{N})$ is the source flow vector, and *S_k_* is the flow from outside to node *k*.

$T=(T_{1},T_{2},\cdots,T_{N}), T_{k}=S_{k}+\sum_{j=1}^{N} f_{jk}=D_{k}+\sum_{j=1}^{N} f_{kj}$, (2)

*T_i_* is the throughflow of i.

$M=\left\{m_{ij}\right\}_{N\times N}$, each element $m_{kj}=\frac{f_{kj}}{T_{k}}$,

M is the Markov chain derived by F.

$U=I+M+M^{2}+\cdots=(I-M)^{-1}$ (3)

Is the fundamental matrix in input-output analysis.

$M'=M-\Delta M$, where, $(\Delta M)_{kj}=\left\{\begin{matrix} m_{kj} if j=i \\ 0 if j\neq i \end{matrix}\right.$, (4)

M’ is the perturbed Markov chain after node i is deleted.

$U'=I+M'+M'^{2}+\cdots=(I-M')^{-1}$ (5)

U’ is the new fundamental matrix after the deletion of i.

$S'=(S_{1}',S_{2}',\cdots,S_{N}')$, where, $S_{k}'=\left\{\begin{matrix} 0 if k=i \\ S_{k} if k\neq i \end{matrix}\right.$

S’ is the influxes from the source after i is deleted.

$T'=S'U'$ (6)

$F'=\left(\begin{matrix} T_{1}' & & & \\ & T_{2}' & & \\ & & \ddots& \\ & & & T_{N}' \end{matrix}\right)\cdot M'$ (7)

F’ is the flux matrix after the deletion of i, it is derived from the balancing condition of the new network.

Using this notation, we will prove a lemma.

**Lemma 1. The equation $u'_{kj}=\frac{u_{kj}}{u_{kk}}$holds for any** $k,j\in[1,N]$

**Proof:**

According to the definitions,

$U=(I-M)^{-1}$ and $U'=(I-M')^{-1}$ (8)

Thus,

$U(I-M)=I=U'(I-M')$ (9)

According to the definition of $M'=M-\Delta M$*,* we have:

$U-U'=U\Delta MU'$ (10)

Thus, the *kth* row and *jth* column of the previous equation should also be hold:

$u_{kj}-u'_{kj}=u'_{kj}\sum_{s=1}^{N} u_{ks}m_{ks}$ (11)

Because: $UM=U-I$, thus:

$\sum_{s=1}^{N} u_{ks}m_{sk}=u_{kk}-1$ (12)

Therefore,

$u_{kj}-u'_{kj}=u'_{kj}(u_{kk}-1)$ (13)

Thus,

$u'_{kj}=\frac{u_{kj}}{u_{kk}}$ (14)

**■**

Then, we can state and proof the following theorem (Theorem 1).

**Theorem1. $C_{i}=\sum_{k=1}^{N} \sum_{j=1}^{N} (S_{j}u_{ji}/u_{ii})u_{ik}=(1,1,\cdots,1)\cdot(T-T')$ holds for any balanced flow network *F***

**Proof:**

According to the flow balancing equations

$\begin{matrix} TM+S=T \\ T'M'+S'=T' \end{matrix}$ (15)

Their difference is:

$TM-T'M'+S-S'=T-T'$ (16)

According to the definitions of M’ and S’,

$T(M'+\Delta M)-T'M'+S-S'=(T-T')M'+T\Delta M+S-S'=T-T'$ (17)

Then:

$T-T'=(T\Delta M+S-S')(I-M')^{-1}$ (18)

We know that $S-S'=(0,0,\cdots,S_{i},0,\cdots,0)$, and $T\Delta M=\sum_{j=1}^{N} T_{j}m_{ji}$, thus:

$T\Delta M+S-S'=(0,0,\cdots,\sum_{j=1}^{N} T_{j}m_{ji}+S_{i},0,\cdots,0)=(0,0,\cdots,T_{i},0,\cdots,0)$ (19)

Thus,

$T-T'=(T\Delta M+S-S')(I-M')^{-1}=T_{i}(u'_{i,1},u'_{i,2},\cdots,u'_{i,N})$ (20)

Because,$T_{i}=\sum_{j=1}^{N} S_{j}u_{ji}$, and Lemma 1,

$(T-T')_{k}=(\sum_{j=1}^{N} S_{j}u_{ji})u'_{i,k}=(\sum_{j=1}^{N} S_{j}u_{ji})\frac{u_{ik}}{u_{ii}}$ (21)

Finally, taking the summation for *k*, we obtain the following:

$(\sum_{k=1}^{N} \sum_{j=1}^{N} S_{j}u_{ji}\frac{u_{ik}}{u_{ii}}=\sum_{k=1}^{N} (T-T')_{k}=(1,1,\cdots,1)\cdot(T-T')$ (22)

■

**References:**

[1]Jiang Zhang, Liangpeng Guo, Scaling behaviors of weighted food webs as energy transportation networks, Journal of Theoretical Biology.264 (2010) , pp:760–770

[2] Hidalgo CA, Klinger B, Barabsi AL, Hausmann R (2007) The product space conditions the development of nations. Science 317: 482–487.

[3] Edward E, 1984: Leamer, Sources of International Comparative Advantage: Theory and Evidence, The MIT Press,
